# Supplementary material for: The active zone protein Clarinet regulates synaptic sorting of ATG-9 and presynaptic autophagy
Source: PLoS Biol. 2023 Apr 13;21(4):e3002030. doi: 10.1371/journal.pbio.3002030 (PMC10101500; doi:10.1371/journal.pbio.3002030)
Supplement: S1 Table — List of all alleles identified in 3 semiclonal forward genetic screens and categorized by phenotypic class. We focused our study on the ola285 allele due to its phenotype (affects ATG-9 localization but does not affect synaptic vesicle protein localization in Zone 2 synapses) and the higher penetrance and expressivity of its phenotype (see Fig 1). (DOCX) [file pbio.3002030.s002.docx]

| NO. | Alleles | Phenotypes | Penetrance |
| --- | --- | --- | --- |
| 1 | *ola285* | ATG-9 localizes to abnormal subsynaptic foci, while RAB-3 is not affected | More than 60% |
| 2 | *ola306* | ATG-9 localizes to abnormal subsynaptic foci, while RAB-3 is not affected | More than 30% |
| 3 | *ola307* | ATG-9 localizes to abnormal subsynaptic foci, while RAB-3 is not affected | Less than 30% |
| 4 | *ola310* | ATG-9 localizes to abnormal subsynaptic foci, while RAB-3 is not affected | Less than 30% |
| 5 | *ola319* | ATG-9 is dim at both the cell body and the neurite | Not scored |
| 6 | *ola320* | ATG-9 is dim at both the cell body and the neurite, while RAB-3 is disorganized at the neurite | Not scored |

**S1 Table. Annotated list of alleles from forward genetic screen.** List of all alleles identified in three semi-clonal forward genetic screens, and categorized by phenotypic class. We focused our study on the *ola285* allele due to its phenotype (affects ATG-9 localization, but does not affect synaptic vesicle protein localization in Zone 2 synapses) and the higher penetrance and expressivity of its phenotype (see Figure 1).
