# Supplementary material for: The active zone protein Clarinet regulates synaptic sorting of ATG-9 and presynaptic autophagy
Source: PLoS Biol. 2023 Apr 13;21(4):e3002030. doi: 10.1371/journal.pbio.3002030 (PMC10101500; doi:10.1371/journal.pbio.3002030)
Supplement: S2 Table — (DOCX) [file pbio.3002030.s003.docx]

| **REAGENT or RESOURCE** | | **SOURCE** | **IDENTIFIER** |
| --- | --- | --- | --- |
| **Chemicals** | | | |
| Levamisole Hydrochloride | Sigma-Aldrich | | 31742 |
| Ethyl methanesulfonate (EMS) | Sigma-Aldrich | | M0880 |
| Muscimol | Abcam | | ab120094 |
| **Experimental Models: Organisms/Strains** | | | |
| *C. elegans*: *olaIs34* [pttx-3::atg-9::gfp+ pttx-3::mCh::rab-3] III | [1] | | DCR4755 |
| *C. elegans*: *unc-101(m1)* I | Caenorhabditis Genetics Center | | DR1 |
| *C. elegans*: *unc-101(m1);olaIs34* | [1] | | DCR8064 |
| *C. elegans*: *unc-101(m1);cla-1(ola285);olaIs34* | This paper | | DCR8065 |
| *C. elegans*: *olaEx4911* [pttx-3::UNC-101]*;unc-101(m1);cla-1(ola285);olaIs34* | This paper | | DCR8091 |
| *C. elegans*: *olaEx5021* [pttx-3::mouse AP-1μ1];*unc-101(m1);cla-1(ola285);olaIs34* | This paper | | DCR8296 |
| *C. elegans*: *olaEx5023* [pttx-3::mouse AP-2μ1];*unc-101(m1);cla-1(ola285);olaIs34* | This paper | | DCR8298 |
| *C. elegans*: *unc-101(sy108)* I | Caenorhabditis Genetics Center | | PS529 |
| *C. elegans*: *unc-101(sy108);olaIs34* | This paper | | DCR8210 |
| *C. elegans*: *unc-101(sy108);cla-1(ola285);olaIs34* | This paper | | DCR8211 |
| *C. elegans*: *dpy-23(e840)* X | Caenorhabditis Genetics Center | | CB840 |
| *C. elegans: dpy-23(e840);olaIs34* | [1] | | DCR8633 |
| *C. elegans: dpy-23(e840);cla-1(ola285);olaIs34* | This paper | | DCR8003 |
| *C. elegans: unc-101(m1);dpy-23(e840); olaIs34* | This paper | | DCR8634 |
| *C. elegans*: *unc-26(s1710)* IV | Caenorhabditis Genetics Center | | EG3027 |
| *C. elegans*: *unc-26(s1710);olaIs34* | [1] | | DCR6121 |
| *C. elegans*: *unc-11(e47)* I | Caenorhabditis Genetics Center | | CB47 |
| *C. elegans*: *unc-11(e47);olaIs34* | [1] | | DCR5893 |
| *C. elegans*: *unc-101(m1);unc-11(e47);olaIs34* | This paper | | DCR8205 |
| *C. elegans*: *sdpn-1(ok1667);unc-11(e47);olaIs34* | This paper | | DCR8313 |
| *C. elegans*: *cla-1(ola285)* | This paper | | DCR8019 |
| *C. elegans*: *cla-1(ola285);olaIs34* | This paper | | DCR8014 |
| *C. elegans*: *unc-18(e81)* X | Caenorhabditis Genetics Center | | CB81 |
| *C. elegans*: *unc-18(e81)*;*olaIs34* | [1] | | DCR8033 |
| *C. elegans*: *unc-18(e81);cla-1(ola285);olaIs34* | This paper | | DCR8034 |
| *C. elegans*: *unc-13(s69)I* | Erik Jorgensen Laboratory (University of Utah, Salt Lake City, Utah) | | BC168 |
| *C. elegans*: *unc-13(s69);olaIs34* | [1] | | DCR8011 |
| *C. elegans*: *unc-13(s69);cla-1(ola285);olaIs34* | This paper | | DCR8002 |
| *C. elegans*: *unc-10(md1117)* X | Caenorhabditis Genetics Center | | NM1657 |
| *C. elegans*: *unc-10(md1117);olaIs34* | This paper | | DCR8031 |
| *C. elegans*: *unc-10(md1117);cla-1(ola285);olaIs34* | This paper | | DCR8032 |
| *C. elegans*: *unc-2(e55)X* | Caenorhabditis Genetics Center | | CB55 |
| *C. elegans*: *unc-2(e55);olaIs34* | This paper | | DCR8203 |
| *C. elegans*: *unc-2(e55);cla-1(ola285);olaIs34* | This paper | | DCR8204 |
| *C. elegans*: *epg-9(bp320)* IV | Caenorhabditis Genetics Center | | HZ1692 |
| *C. elegans*: *epg-9(bp320);olaIs34* | [1] | | DCR7047 |
| *C. elegans*: *epg-9(bp320);cla-1(ola285);olaIs34* | This paper | | DCR8480 |
| *C. elegans*: *olaIs35* [pttx-3::egfp::lgg-1;pttx-3::mCh] | [2] | | DCR4750 |
| *C. elegans*: *cla-1(ok560)* IV | Caenorhabditis Genetics Center | | RB778 |
| *C. elegans*: *cla-1(ok560);olaIs34* | This paper | | DCR6126 |
| *C. elegans*: *cla-1(ok560);olaIs35* | This paper | | DCR7296 |
| *C. elegans*: *cla-1(ola285);olaIs35* | This paper | | DCR8596 |
| *C. elegans*: *ehs-1(ok146)II* | Caenorhabditis Genetics Center | | NM1568 |
| *C. elegans*: *ehs-1(ok146);olaIs34* | This paper | | DCR8071 |
| *C. elegans*: *ehs-1(ok146);cla-1(ola285);olaIs34* | This paper | | DCR8234 |
| *C. elegans*: *itsn-1(ok268)IV* | Caenorhabditis Genetics Center | | VC201 |
| *C. elegans*: *itsn-1(ok268);olaIs34* | This paper | | DCR8072 |
| *C. elegans*: *itsn-1(ok268);cla-1(ola285);olaIs34* | This paper | | DCR8235 |
| *C. elegans*: *sdpn-1(ok1667)X* | Caenorhabditis Genetics Center | | RB1460 |
| *C. elegans*: *sdpn-1(ok1667);olaIs34* | This paper | | DCR8236 |
| *C. elegans*: *sdpn-1(ok1667);cla-1(ola285);olaIs34* | This paper | | DCR8237 |
| *C. elegans*: *cla-1(wy1048)* | [3] | | DCR5584 |
| *C. elegans*: *cla-1(wy1048);olaIs34* | This paper | | DCR5985 |
| *C. elegans*: *cla-1(ola324)* [floxed cla-1L] | [3] | | DCR5545 |
| *C. elegans*: *cla-1(ola324)* [floxed cla-1L]*; olaIs34* | This paper | | DCR6395 |
| *C. elegans*: *olaEx3808* [pmod-1::nCRE]*; cla-1(ola324)* [floxed cla-1L]; *olaIs34* | This paper | | DCR6433 |
| *C. elegans*: *olaEx4099* [pttx-3::sng-1::bfp];*olaIs34* | [1] | | DCR6894 |
| *C. elegans*: olaEx4099; *cla-1(ola285);olaIs34* | This paper | | DCR8482 |
| *C. elegans*: *olaEx4060* [pttx-3::atg-9::mCh+ pttx-3::sng-1::gfp] | [1] | | DCR6797 |
|  |  | |  |
| *C. elegans*: *cla-1(ola285);olaEx4060* | This paper | | DCR8481 |
| *C. elegans*: *olaEx4290* [pttx-3::bfp::chc-1];*olaIs34* | [1] | | DCR7140 |
| *C. elegans*: *olaEx4290;cla-1(ola285);olaIs34*  *C. elegans: cla-1(ola506)IV[GFP::CLA-1L];cla-1(ola285)* | This paper  This paper | | DCR8774  DCR9025 |
| *C. elegans*: *cla-1(ola311)IV* [GFP::CLA-1L] | [3] | | DCR5409 |
| *C. elegans*: *cla-1(ola311); olaEx5236* [punc129::apt-4::mCh] | This paper | | DCR5409 |
| *C. elegans: cla-1(wy1186)IV* [C-terminal FRT-stop-FRT GFP] | [3] | | TV23058 |
| *C. elegans: cla-1(wy1186);olaex5237* [punc-129::apt-4::mCh+prab-3::FLPase] | This paper | | DCR8698 |
| *C. elegans: wyEx1826* [pttx-3::gfp::syd-2] | [2] | | TV4699 |
| *C. elegans: cla-1(ok560);wyEx1826*  *C. elegans: olaEx2264* [punc-14::atg-9::GFP]  *C. elegans:* cla-1(ola285);olaEx2264 | This paper  [2]  This paper | | TV4699  DCR4535  DCR8940 |
| **Recombinant DNA** | | | |
| Plasmid: pttx-3::atg-9::gfp | [2] | | DACR808 |
| Plasmid: pttx-3::sng-1::bfp | [1] | | DACR2739 |
| Plasmid: pttx-3::mCh::rab-3 | [4] | | DACR18 |
| Plasmid: pttx-3::sng-1::gfp | [1] | | DACR2716 |
| Plasmid: pttx-3::mCh | [2] | | DACR63 |
| Plasmid: pttx-3::gfp::lgg-1 | [2] | | DACR1321 |
| Plasmid: pttx-3::bfp::chc-1 | [1] | | DACR2877 |
| Plasmid: pmod-1::nCRE | This paper | | DACR2185 |
| Plasmid: punc-129::apt-4::mCh | [5] | | KP-JB983 |
| Plasmid: prab-3::FLPase | This paper | | DACR3506 |
| Plasmid: pttx-3::UNC-101 | This paper | | DACR3473 |
| Plasmid: pttx-3::mouse AP-1μ1 | This paper | | DACR3533 |
| Plasmid: pttx-3::mouse AP-2μ1 | This paper | | DACR3534 |
| Plasmid: pttx-3::gfp::syd-2 | [2] | | DACR430 |
| **Software and Algorithms** | | | |
| Volocity | Improvision by Perkin Elmer | | N/A |
| Fiji | [6] | | <https://imagej.net/Fiji/Downloads> |
| Prism | Graphpad Software Inc | | <https://www.graphpad.com/> |
| Adobe Illustrator | Adobe | | <https://www.adobe.com/products/illustrator.html> |
| ApE |  | | https://jorgensen.biology.utah.edu/wayned/ape/ |
| **Other** | | | |
| UltraView VoX spinning disc confocal microscope with a 60x CFI Plan Apo VC, NA 1.4, oil objective on a NikonTi-E stand | PerkinElmer | | N/A |

Hammamatsu C9100-50 camera Hammamatsu N/A

**S2 Table. Key resources table.**

1. Yang S, Park D, Manning L, Hill SE, Cao M, Xuan Z, et al. Presynaptic autophagy is coupled to the synaptic vesicle cycle via ATG-9. Neuron. 2022;110(5):824-40 e10. Epub 2022/01/24. doi: 10.1016/j.neuron.2021.12.031. PubMed PMID: 35065714; PubMed Central PMCID: PMCPMC9017068.

2. Stavoe AKH, Hill SE, Hall DH, Colon-Ramos DA. KIF1A/UNC-104 Transports ATG-9 to Regulate Neurodevelopment and Autophagy at Synapses. Dev Cell. 2016;38(2):171-85. doi: 10.1016/j.devcel.2016.06.012. PubMed PMID: WOS:000380825000011.

3. Xuan Z, Manning L, Nelson J, Richmond JE, Colon-Ramos DA, Shen K, et al. Clarinet (CLA-1), a novel active zone protein required for synaptic vesicle clustering and release. Elife. 2017;6. Epub 2017/11/22. doi: 10.7554/eLife.29276. PubMed PMID: 29160205; PubMed Central PMCID: PMCPMC5728718.

4. Colon-Ramos DA, Margeta MA, Shen K. Glia promote local synaptogenesis through UNC-6 (netrin) signaling in C-elegans. Science. 2007;318(5847):103-6. doi: 10.1126/science.1143762. PubMed PMID: WOS:000249915400051.

5. Bai JH, Hu ZT, Dittman JS, Pym ECG, Kaplan JM. Endophilin Functions as a Membrane-Bending Molecule and Is Delivered to Endocytic Zones by Exocytosis. Cell. 2010;143(3):430-41. doi: 10.1016/j.cell.2010.09.024. PubMed PMID: WOS:000283603900016.

6. Schindelin J, Arganda-Carreras I, Frise E, Kaynig V, Longair M, Pietzsch T, et al. Fiji: an open-source platform for biological-image analysis. Nat Methods. 2012;9(7):676-82. Epub 2012/06/30. doi: 10.1038/nmeth.2019. PubMed PMID: 22743772; PubMed Central PMCID: PMCPMC3855844.
