# Supplementary material for: The active zone protein Clarinet regulates synaptic sorting of ATG-9 and presynaptic autophagy
Source: PLoS Biol. 2023 Apr 13;21(4):e3002030. doi: 10.1371/journal.pbio.3002030 (PMC10101500; doi:10.1371/journal.pbio.3002030)
Supplement: Attachment — Submitted filename: Rebuttal Letter_Xuan PLoS.docx [file pbio.3002030.s017.docx]

*Daniel A. Colón-Ramos, McConnell Duberg Professor*

*Department of Neuroscience and Cell Biology*

*Program in Cellular Neuroscience, Neurodegeneration and Repair (CNNR)*

*Yale University School of Medicine*

*260 Whitney Avenue, YSB C167*

*New Haven, Connecticut 06511*

*Telephone: 203 737-3438*

*Email: daniel.colon-ramos@yale.edu*

Yale University

November 14, 2022

Dear Dr. Kris Dickson:

Per our conversation at the CSH conference, enclosed please find our revised manuscript “**The active zone protein Clarinet regulates synaptic sorting of ATG-9 and presynaptic autophagy**” and our responses to the reviews received from Developmental Cell. We have added new data and edits which address all major points raised by the reviewers. Briefly, the reviewers had two major concerns:

1. A better understanding of the CLA-1 phenotype via super-resolution or electron microscopy. To address this, our revised manuscript now includes electron microscopy reconstructions of presynaptic sites, quantification of the distribution of organelles at presynaptic regions and immunogold electron microscopy to determine ATG-9::GFP localization. These studies were conducted for both *cla-1* mutants and wild type animals, and performed by sectioning animals and identifying the presynaptic site of the AIY interneuron, a technical feat that allowed us to rigorously compare the electron microscopy with the light microscopy studies for a single neuron in an intact animal. Our electron microscopy studies bolster our light microscopy findings, demonstrating that in *cla-1* mutants, ATG-9 abnormally concentrates at vesicular structures in presynaptic regions.
2. A better distinction between the contributions presented in this study and the Yang *et al*. manuscript from our lab (at the time of review, in BioRxiv). The reviewers pointed out that *“it is difficult appreciate some of the logic without recourse to a manuscript that has not yet undergone peer review”*. Yang *et al* has since undergone peer-review and was published in *Neuron.* Additionally and building on the reviewer’s suggestions, in this revised version of the manuscript we have re-written the abstract, introduction and discussion to better underscore the significance of our findings in the context of the literature, including emphasizing that:
   a. Our findings underscore the importance of active zone proteins in regulating local sorting of autophagy proteins and presynaptic autophagy.
   b. Our study uncovers mechanisms that genetically cooperate with active zone proteins in sorting of ATG-9 at synapses.
   c. Our study reveals that sorting of ATG-9 and synaptic vesicle proteins is genetically separable, supporting that *in vivo,* ATG-9 containing vesicles represent a distinct subpopulation of vesicles at synapses.
   d. Our study provides a mechanistic framework to understand how structural components of the synapses, such as active zone proteins, can regulate synaptic autophagy by coupling synaptic vesicle cycle with sorting of autophagic proteins such as ATG-9.

We also integrated the textural clarifications, quantifications, and imaging requests made by the reviewer. We believe this revised manuscript is much improved, and present it for publication in *PloS Biology*.

Daniel A. Colón-Ramos, PhD

Professor of Neuroscience and Cell Biology

**REVIEWER COMMENTS**

**Reviewer 1.**

*The manuscript by Xuan et al. investigates the role of the active zone protein Clarinet in regulating ATG-9 trafficking at synapses and presynaptic autophagy. The authors identified Clarinet in a genetic screen for mutants disrupting ATG-9 localization, and follow this observation up by testing the effects of other known endocytic trafficking proteins on ATG-9 localization. Then they test the effects of Clarinet mutants on activity-dependent presynaptic autophagy. Based on these observations, they propose a model in which the exo/endocytosis of ATG-9 regulates activity-dependent autophagy at the synapse.*

*There are a number of strengths to this manuscript, including the expertise of the lab, the many mutants tested, and the thoughtful hypotheses that are investigated. There are also significant weaknesses to the manuscript in its present form that should be addressed. These include issues with the strength of the premise, issues with some of the data analysis, lack of precision in the description of many of the observations, and lack of integration with previously published work. Together, these weaknesses mean that the work is not yet ready for publication. Specific points to consider:*

**R1.1:** **The manuscript builds very heavily on unpublished work from the same lab. It is difficult to appreciate some of the logic without recourse to a manuscript that has not yet undergone peer review. More significantly, the two manuscripts overlap conceptually, so it is difficult to independently judge the advances reported in this work without considering the advances that should be considered germane to Yang et al. on biorxiv.**

While both the *Yang et al* and *Xuan et al* studies emerge from efforts in our lab to examine ATG-9 trafficking and sorting at synapses, they represent independent studies with unique (but synergistic) contributions. Yet, we can now see how, because of the preprint nature of the *Yang et al* study at the time and because of how we originally framed this study, it would have been difficult for readers to differentiate the relative contributions of the two studies, and appreciate the opportunity for clarification. The *Yang et al* study is now peer-reviewed and published. We edited the abstract, introduction and discussion to better frame the significance of this study in the context of that article and the field.

Briefly, and as now presented in the introduction, ATG-9 trafficking regulates local autophagy in different cellular contexts. In neurons, ATG-9 is transported to presynaptic sites where, like synaptic vesicles, ATG-9-containing vesicles undergo activity-dependent exo-endocytosis. This is regulated by canonical synaptic molecules such as endophilin A and synaptojanin 1, a finding we presented in (Yang et al., 2022). Yet, beyond the requirement of endophilin A and synaptojanin 1, the mechanisms that sort of ATG-9 at presynaptic sites, the relationship of these mechanisms to those that sort canonical synaptic vesicle proteins and the relationship between ATG-9 and the synaptic machinery is not well understood. Knowledge of the molecules required for specific sorting of ATG-9 at synapses is of critical importance to understand the mechanisms that regulate synaptic autophagy, and was the focus of this study. We better explain and discuss how through unbiased forward genetic screens and cell biological studies, we uncover molecules of synaptic machinery, including active zone protein *cla-1*, specifically involved in ATG-9 sorting. In this revised version, we also add new electron microscopy data (performed in intact organisms) to support our phenotypic characterizations of synapses in *cla-1* mutants (new Figures 2, 3 and Supplementary Figure 3).

**2. One of the major points raised here is that mutations in Clarinet lead to accumulation of ATG-9 into clathrin-rich endocytic intermediates, but I had trouble identifying the data clearly showing this point.**

We addressed the concern in two ways:

1. In the original manuscript, these data were in the Supplementary Figure 2. We moved images of ATG-9 colocalization with the Clathrin subunit *chc-1* to new Figure 5 A-H. We also quantified the colocalization between ATG-9::GFP and CHC-1::BFP (new Figure S2).
2. To better understand the ATG-9 phenotype in *cla-1*, we performed transmission electron microcopy studies in which we reconstructed and quantified the morphology of synapses in wild-type and *cla-1* mutant animals. We also performed immuno-EM and identified ATG-9 containing vesicular structures (new Figures 2 and 3). We found that while mutation in *cla-1L* does not result in gross abnormalities in synaptic morphology, ATG-9::GFP gold particles were abnormally concentrated on subsynaptic regions. Our studies support our light microscopy observations that ATG-9 containing structures abnormally form subsynaptic foci. In the discussion, we elaborate about how this abnormal ATG-9 phenotype is reminiscent to that observed in vertebrate cells with defective autophagy (Olivas et al., 2022).

**3. The authors frequently refer to defects in ATG-9 "trafficking", when what they are scoring is "localization". In the mutants, the observation is that ATG-9 is mislocalized, and the interpretation is that this is due to a trafficking defect. But in single time point assays and in the absence of more dynamic data showing trafficking is altered, the authors should more clearly distinguish between observations and interpretation.**

We agree that ‘sorting’ is a better term to convey what we are observing and have edited the text appropriately, including the title.

**4. The authors start with a description of a genetic screen for genes regulating the presynaptic localization of ATG-9, but only discuss Clarinet. How many genes did they identify? Were any other hits germane to this story?**

We have now included a table summarizing the mutants (new Table S1) and now also better explain in the text why we focused on allele *ola285*. Briefly, we chose this allele because it phenocopied the sorting defects observed for *unc-26/synaptojanin,* yet, unlike *unc-26* mutants, it did not display defects in synaptic vesicle protein localization in the synaptic Zone 2 region.

**5. The penetrance of the single Clarinet mutation is relatively low - why? In regard to the expressivity of the phenotype, I have significant concerns about the way this is measured. The authors note that they measure ATG-9 intensities using different exposures for WT and mutant animals. This is a non-standard approach that requires sufficient validation, which is not provided in the manuscript. The index approach is also not used consistently; in Figure 1, data on ATG-9 localization is reported as an index, whereas the parallel data on SYD-2 localization in Supplemental Figure 1 is reported as mean intensity. While their index amplifies the extent of the change in localization by dividing the difference between highs and lows by the low value, it may also increase the apparent differences in expressivity, a concern giving the apparent biphasic nature of the phenotype when assessed via the index measurement. Intensity measurements should all be collected with the same settings of exposure time, gain, etc, so the current approach should be reconsidered or more extensively validated. Area measurements might offer a more robust analytic that better describes the clear phenotypic changes shown in the images provided**

The reviewer raises three points which we address separately below:

i. The penetrance of the *cla-1* phenotype was, in our experience, high and clearly distinguishable from wild type counterparts. Even when we used stringent criteria for defining the phenotype (which we do in this paper when we define the penetrance as 60% of observed animals), we were able to easily identify mutants with the phenotypic differences. Perhaps the best explanation of how distinguishable the phenotype is regards the fact that to positionally map the lesion to the *cla-1* gene, we were able to outcross it to different strains, generate recombinant strains for mapping and recover the lesion based on the phenotypic differences.
ii. We used different methods to quantify the phenotypes of ATG-9 and SYD-2 in the wild-type and *cla-1* genotypes to best test the hypotheses examined. For example, the ATG-9 phenotype, the distribution of ATG-9 is different for wild-type animals (diffusely distributed along zone 2) and *cla-1* mutants (enriched in subsynaptic foci). (F_peak_-F_trough_)/F_trough_) was used to score this enrichment. For the SYD-2 phenotype, however, we did not see a difference in the distribution of SYD-2 in Zone 2 between wild-type and *cla-1* mutant animals. We decided to instead measure the level of SYD-2 (all settings for the confocal microscope and camera were kept identical across genotypes for these experiments) to determine if there were detectable differences between the examined genotypes. We underscore that to better examine these hypotheses, we now also include new electron microscopy data which bolsters our findings for the conclusions reached in both of these experiments (Figures 2 and 3).
iii. Regarding the imaging parameters used during the quantifications of ATG-9 expressivity, we decided to image in the dynamic range to avoid saturation and properly capture the distribution of signal across the synapse for the parameter index used (F_peak_-F_trough_)/F_trough_) and across genotypes. We note that although this was done out of caution, we did validate these parameters for wild type animals and observed that, while it increased the distribution, the index was consistent across different imaging confocal settings used in this study and did not change out conclusions or results (Panel J below).

**6. As the authors note multiple times throughout the text, Clarinet has similarities to Piccolo and Bassoon, and Bassoon has been reported to also effect synaptic autophagy. Yet the authors do not address the similarities/differences in a satisfying way.**

We have now added a paragraph in the discussion comparing these active zone proteins in the context of their roles in autophagy. Briefly, we find CLA-1 acts via a different mechanism than that reported for Bassoon and Piccolo. We underscore that, despite the mechanistic differences, together these studies support the concept that in neurons, active zone proteins play important roles in regulating of activity-dependent synaptic autophagy.

**7. The images comparing ATG-9, SNG-1, and RAB-3 localization are interesting, but the images appear overexposed so that it is unclear if there is actual co-localization among any of these proteins or just co-enrichment. For example, SNG-1 appears more punctate and ATG-9 more extensively distributed, but it is hard to be sure from the images provided.**

We carefully adjusted the confocal settings for each channel to avoid saturation (see comment 5). To better present the colocalization relationships among different channels, we choose "channel first then z-stack mode". We observe that the two synaptic vesicle proteins (SNG-1 and RAB-3) colocalize with each other better than each of them with ATG-9 in both wild-type (see new Figure 2A-D) and *cla-1* mutant (see Figure new 2E-H) animals. We include a Pearson coefficient of localization (Supplementary Figure 2B) to compare co-localization of ATG-9 and SNG-1 or CHC-1 in *cla-1* mutants. We now also include new electron microscopy data which provide new insights regarding the relationship between ATG-9 containing structures and synaptic vesicles (Figures 2 and 3).

**8. The authors address effects on autophagy in a single figure, but here again the quality of the images raises concern. From the single image of LGG-1 shown in Figure 8 (panel A), it is unclear how they can unambiguously measure puncta number**

We agree that it is challenging to convey, in that single image, the observed phenotypes. We now therefore better explain in the text how we have verified GFP::LGG-1 as markers for autophagosomes in AIY. Briefly, we have determined in previous studies that GFP::LGG-1(G116A), which contains a point mutation that prevents LGG-1 lipidation and conjugation to autophagosomes, does not form puncta. Furthermore, we crossed GFP::LGG-1 into mutants in early autophagy and late autophagy and found the LGG-1 puncta number decreased in early autophagy mutants and increased in late autophagy mutants as expected (Stavoe et al., 2016) and as confirmed by electron microscopy studies (Hill et al., 2019), which gives us confidence that GFP::LGG-1 puncta represent autophagosomes. For this study, counting of LGG-1 puncta was performed by two researchers, independently and blinded to genotypes.

**9. Also in Figure 8, it is surprising that there is no effect on baseline autophagy, only temperature-induced autophagy. Why?**

We have consistently seen, both in this study and in (Yang et al., 2022), a genetic separation between baseline autophagy and activity induced autophagy. We speculate that baseline autophagy is maintained through other mechanisms that do not require the pathways characterized in this study (now in the discussion).

**10. In sum, this work addresses a potentially important question, but the presentation is too preliminary as of yet. Cleaner, more rigorous imaging data, more straightforward imaging analysis, clearer distinctions between experimental observations and likely interpretations would improve the technical aspects of the work, while further distinguishing this work from the biorxiv manuscript by the same group and further comparisons to the previously published bassoon work would strengthen the scientific impact of the study.**

We appreciate the comments of the reviewer and believe that the reframing in this edited version and the new electron microscopy data significantly strengthens the paper and clarifies its contributions.

**Reviewer 2.**

*This manuscript describes experiments to better understand how ATG-9 "trafficking" at synapses is regulated. This is of some interest as ATG-9 is a component of the autophagy machinery shown to be both present and functionally relevant to presynaptic function. In their study, the authors performed a screen in C. elegans to that identified the presynaptic active zone protein Clarinet as a regulator of ATG-9 trafficking. Utilizing a collection of exocytic and endocytic mutants and fix tissue imaging, the authors also show that mutations in the long form of Clarinet (CLA-1L) leads to the accumulation of ATG-9::GFP in large clathrin positive puncta, presumably an endocytic intermediate. Their genetic analysis supports that these structures are formed from early endocytic membranes requiring Syndapin, AP2/AP180, EPS15 and ITSN-1 as well as Clarinet. While the recycling of synaptic vesicle proteins also requires these proteins, loss of function in CLA-1L selectively appears to disrupt the trafficking of ATG-9, implying that its N-terminal repetitive region is require for this specificity. Of note, the disrupted trafficking of ATG-9 in cla-1(ola285) mutants also disrupts activity induced autophagy. Conversely, disruptions in autophagy, also leads to the accumulation of ATG-9::GFP in boutons. These latter data imply an interrelationship between ATG-9 trafficking and autophagy. On many levels the data presented in this manuscript are of a high quality and generally support the conclusions of the authors. Particularly impactful is the identification of Clarinet in this process. There are nonetheless several limitations that require some attention.*

**1. First the author's repeated site a paper submitted to bioRxiv in 2020 (Yang et al.) entitled "Presynaptic autophagy is coupled to the synaptic vesicle cycle via ATG-9". This is problematic as many of the same type of experiments with similar conclusions that are in the current manuscript are also present there, albeit with a slightly different emphasis that does not involve Clarinet. The authors should clarify this issues as it greatly reduces the impact and originality of the current paper. Importantly, many of the key claims made in the current manuscript are made in that paper (e.g. the role of exo and endocytosis as well as proteins of the endocytic machinery clathrin etc and autophagy in the trafficking of ATG-9). One crucial one is the statement that ATG-9 couples autophagy by its association with the synaptic vesicle cycle. Yet the evidence that this is true is solely based on genetics and not the functional recycling of an ATG-9 associated SV.**

This is a point also raised by R1C1, and we appreciate the opportunity for clarification. To address this, in the revised study we edit the abstract, introduction and discussion to underscore the importance of this study in the context of the field, including the *Yang et al* study (now published).

As we now better explain, in the unbiased forward genetic screen that resulted in the identification of CLA-1, we sought to identify alleles that differentially affected ATG-9 localization from synaptic vesicle protein localization. We designed the screen this way as to understand three outstanding questions in the field: how is ATG-9 sorted at presynaptic sites? what is the relationship of the mechanisms that sort ATG-9 to those that sort canonical synaptic vesicle proteins? what is the relationship between ATG-9 containing vesicles and synaptic vesicles? In this edited version we better explain how, by conducting forward genetic screens at single synapses of *C. elegans* neurons, we advance our understanding of these questions by identifying an unexpected role for active zone protein Clarinet (CLA-1L) in sorting of autophagy protein ATG-9 at synapses, and presynaptic autophagy. We also explain, and better discuss, that our study uncovers molecules and synaptic machinery specifically involved in ATG-9 sorting, and underscores the importance of active zone proteins in regulating local sorting of autophagy proteins and presynaptic autophagy. Lastly, we demonstrate that our findings support a model whereby CLA-1L bridges the exocytic active zone regions with the endocytic periactive zone to regulate presynaptic sorting of ATG-9.

We also add new electron microscopy data (performed in intact organisms) that, along our genetic and cell biological studies, support that ATG-9 containing vesicles represent a distinct subpopulation of vesicles at synapses, and discuss the implications of this findings.

**2. The issue of ATG-9 and SVs is yet more problematic based on claims in the current paper that ATG-9 is recycled within boutons during activity as an integral membrane protein within SVs. Given the specificity of the cla-1(ola285) mutation, the authors push this concept further stating that during its recycling through endosome it forms its own SVs. If true this is no trivial observation, as the existence of subspecies of SVs, while purported, have not been convincingly documented. Moreover, it has also been argued that the membranes that create autophagosome within in boutons are derived locally, either from the endosomal, plasma membrane and/or membranes from SVs. Thus, should ATG-9 "vesicle" really exist and recycle as suggested in this manuscript, this could be fundamental to the field, increasing the impact of this manuscript. One strategy to address both of these concerns a) overlap with the Yang paper and b) evidence that ATG-9 is indeed on a subspecies of SVs that directly contribute to presynaptic autophagy, is to include more direct evidence that the latter was true. To this end, there are several key experiments that should to be preform for this manuscript to meet the caliber of Dev Cell. First, there needs to more direct evidence that ATG-9 is on a ~50nm SV-like vesicle. This could/should include biochemical, super-resolution microscopy and Immuno-EM (as performed in the Yang paper) data. The latter two could be performed in Zone 3. Of note, the minimally overlapping patterns of Rab3 and ATG-9 in Zone3 Fig 1B-D of this paper, where these vesicles are in transit, could be a good place to explore this uniqueness as well as the dimension of ATG-9 vesicles.**

As suggested by the reviewer, we performed transmission EM and Immuno-EM in wild type and *cla-1(ola285)* mutants (new Figure 2 and 3). Our electron microscopy studies were performed in single, identifiable neurons of intact animals, a technical feat which allowed comparisons with our fluorescent microscopy observations. Our findings bolster our understanding of the ATG-9 phenotype in *cla-1L* mutant animals and the nature of the ATG-9-containing vesicles at the synapse, which we discuss in the context of the literature in the revised manuscript. We underscore (and now discuss in the text) that the EM phenotype we observe for ATG-9-containing vesicular structures in *cla-1* mutants are reminiscent to those seen in vertebrate cells during defective autophagy (Olivas et al., 2022), supporting our conclusions on the role of *cla-1* in ATG-9 sorting and regulation of local autophagy in neurons.

**3. Second, it is suggested that the authors create and test a pHluorin version of ATG-9 to formally show that ATG-9 associated presynaptic vesicle recycle with the plasma membrane in an activity dependent manner. This would complement genetic studies using unc-13, unc-10 and unc-18 mutation, which in and of themselves reveal only that "something" must recycle with the plasma membrane to bring ATG-9 into clathrin-coated structure, not that ATG-9 does so, a challenge that is compounded by the fact given that membrane flow is not unidirectional.**

We agree that visualizing the dynamics of ATG-9 exo-endocytosis with a reagent like pHluorin would be valuable. We explored this possibility, but it is not currently feasible in our system. Briefly, ATG-9 was, until recently, modeled as a six-pass transmembrane protein (Young et al., 2006). Recent structural studies, however, have demonstrated that the human ATG9A is a four transmembrane domain protein with two helices that are partially embedded in the membrane, and with both N-terminal and C-terminal tails in the cytosol (Guardia et al., 2020). Therefore, tagging the protein on the vesicle lumen side could require insertion of the pHluorin into a protein loop. To do this properly, insertion would have to be achieved without disruption the protein function, something we were unable to do.

In the published version of *Yang et al 2022*, however, to better examine ATG-9 exo-endocytosis, we included additional analyses of ATG-9 dynamics in loss-of-function alleles that transiently inhibit endocytosis, as well as chemo-genetic and optogenetic approaches that predictably alter the neuronal activity state of the AIY interneurons. These findings were not in the original *Yang et al* BioRxiv paper. We now edited this manuscript to better discuss the findings in *Yang et al 2022* as well as other published findings in the field.

**Reviewer 3.**

*In this study, Zhao et al report that an unusually large isoform of the presynaptic active zone protein Clarinet (CLA-1L) regulates exo-endocytic trafficking of the autophagy protein ATG-9 in AIY neurons of C. elegans. They provide multiple lines of evidence that CLA-1L acts cell autonomously and specifically to affect presynaptic ATG-9 distribution, not other presynaptic vesicle proteins, such as SNG-1 and RAB-3. They show that in cla-1(L) mutants ATG-9 colocalizes with clathrin heavy chain subunit CHC-1. They carried out a series of genetic analysis to dissect the interactions of CLA-1L with proteins involved in synaptic vesicle docking and fusion, endocytic scaffolding and clathrin adaptor complexes. They draw several conclusions: first, CLA-1L requires factors in synaptic vesicle fusion/exocytosis to regulate ATG-9 subsynaptic accumulation; second, CLA-1L acts cooperatively with the EHS-1-ITSN-1 endocytic scaffolding complex in ATG-9 presynaptic sorting; third, SDPN-1/syndapin 1 and AP-1 adaptor complex, which are involved in activity-dependent bulk endocytosis (ADBE), sort ATG-9 to endocytic intermediates; forth, AP-2 and AP180, which mediated clathrin-mediated endocytosis (CME) of synaptic vesicle proteins, act in parallel with CLA-1L to sort ATG-9 out of the endocytic intermediates. Furthermore, using endogenous reporters labeling different isoforms of CLA-1, they show that CLA-1L is anchored to the active zone and extends to the periactive zone where endocytosis occurs, suggesting a spatially distinctive role of CLA-1L in endocytic sorting of ATG-9. Finally, they find that like ATG-9, CLA-1L is required for activity-induced autophagosome formation. Overall, the genetic analysis is very thorough and supports mechanistic specificity of CLA-1L in regulating presynaptic ATG-9 localization.*

**1. The manuscript is well written, genetic jargons are easy to follow. Two main points could be further strengthened. First, they identified a missense mutation in CLA-1L to alter ATG-9 pattern. It is unclear how this mutation alters CLA-1L and how CLA-1L interacts with ATG-9 at the protein level. For example, do the two proteins bind each other? Does the mutant CLA-1L fail to bind ATG-9?**

To address this concern and better assess the effects of the lesion of allele *ola285* in CLA-1 protein product *in vivo*, we inserted, via CRISPR, a DNA sequence encoding GFP at the 5’-end of the endogenous *cla-1* locus. The inserted GFP specifically labels CLA-1L. We observed that CLA-1L expression levels were drastically reduced in *ola285* as compared to wild-type animals (Figure S1C-E). Since CLA-1L level is drastically reduced, we think the mutation of CLA-1L disrupts ATG-9 sorting by reducing the CLA-1L expression level rather than by changing the binding properties of CLA-1L to ATG-9. Considering the position of the mutation (single T-to-A nucleotide substitution in Exon 15 of *cla-1L* that results in a nonsense mutation I5753N), it is likely that the mutation results in an inappropriate expression and degradation of the protein. The large size of CLA-1L, its large disordered regions and its relatively low abundance (due to its synaptic nature) prevented us from cloning it, purifying it or performing biochemical assays to examine its bindings partners. We believe, however, that the *in vivo* genetic interactions examined in this study represent a significant contribution towards understanding its mechanism in ATG-9 sorting. We add a sentence in the discussion acknowledging the limitation of the study in regards to our biochemical understanding of CLA-1L, and better explain the value of what we do understand regarding the conducted genetic and cell biological studies.

**2.** **Second, almost all genetic interaction studies used ATG-9 localization, or mislocalization in cla-1L mutants, as functional readouts. It is unclear whether such genetic interactions alter autophagosomes at synapses. Minimally, they should examine levels of synaptic autophagosomes, e.g. number of LGG-1, in double mutants of cla-1(L) with each class of synaptic exo-endocytosis genes.**

We focused on examining autophagosomes in *cla-1* *(ola285)* mutants based on the observation that this allele did not affect synaptic integrity, enabling us to differentiate between roles in synaptic function and autophagy. Similar analyses in other synaptic proteins are more complicated to interpret due to the roles of these proteins in synaptic function (inhibition of synaptic function also can affects synaptic autophagy). We have initiated the suggested studies and observed autophagy phenotypes for the examined proteins, as expected, but it will require further characterization in future studies (and similar to those done in *Yang et al 2022*) to dissect the specific roles of these alleles in synaptic function vs synaptic autophagy.

**3. Other minor points:**

- **In a previous study they showed that ATG-9 and the autophagy pathway regulate PVD axon outgrowth. Does cla-1L have any role in PVD?**

We interpret our *cla-1* phenotypes in ATG-9 sorting to be post-developmental, and therefore distinct from the ATG-9 requirement during development. Consistent with this interpretation, in a previous study we characterized the *cla-1* phenotype in the PVD neuron, and did not observe obvious axon outgrowth defects (Xuan et al., 2017). We do agree that it would be interesting in future studies to systematically examine whether and how CLA-1L regulates ATG-9 in different cell types, particularly given that the CLA-1 isoforms are differentially expressed in distinct neuron types (Xuan et al., 2017).

- **Line 165: 'forward genetic screens' suggest multiple screens, is this the case?**

We performed three rounds of forward genetic screens and identified multiple hits from the screens. We now better explain it in the result sections and Table S1.

- **Line 203: please clarify how exactly cla-1(ok560) affects CLA-1L**

*ok560* is a deletion in the promoter and part of the coding region of *cla-1L* ((Xuan et al., 2017) and Supplemental figure 1 in this study)

- **Throughout the manuscript, the authors frequently use the wording 'cla-1 phenotypes', or 'atg-9 phenotype', please be specific what phenotypes are.**

We have edited the manuscript to address this point.

- **Line 288: CLA1 means CLA-1L?**

Yes. Thanks for pointing it out. We now correct the typos.

- **line 374: typo SDNP-1**

Thanks for pointing it out. We now correct the typos.

GUARDIA, C. M., CHRISTENSON, E. T., ZHOU, W., TAN, X. F., LIAN, T., FARALDO-GOMEZ, J. D., BONIFACINO, J. S., JIANG, J. & BANERJEE, A. 2020. The structure of human ATG9A and its interplay with the lipid bilayer. *Autophagy***,** 1-2.

HILL, S. E., KAUFFMAN, K. J., KROUT, M., RICHMOND, J. E., MELIA, T. J. & COLON-RAMOS, D. A. 2019. Maturation and Clearance of Autophagosomes in Neurons Depends on a Specific Cysteine Protease Isoform, ATG-4.2. *Developmental Cell,* 49**,** 251-+.

OLIVAS, T. J., WU, Y., YU, S., LUAN, L., CHOI, P., NAG, S., DE CAMILLI, P., GUPTA, K. & MELIA, T. J. 2022. ATG9 vesicles comprise the seed membrane of mammalian autophagosomes. *bioRxiv***,** 2022.08.16.504143.

STAVOE, A. K. H., HILL, S. E., HALL, D. H. & COLON-RAMOS, D. A. 2016. KIF1A/UNC-104 Transports ATG-9 to Regulate Neurodevelopment and Autophagy at Synapses. *Developmental Cell,* 38**,** 171-185.

XUAN, Z., MANNING, L., NELSON, J., RICHMOND, J. E., COLON-RAMOS, D. A., SHEN, K. & KURSHAN, P. T. 2017. Clarinet (CLA-1), a novel active zone protein required for synaptic vesicle clustering and release. *Elife,* 6.

YANG, S., PARK, D., MANNING, L., HILL, S. E., CAO, M., XUAN, Z., GONZALEZ, I., DONG, Y., CLARK, B., SHAO, L., OKEKE, I., ALMORIL-PORRAS, A., BAI, J., DE CAMILLI, P. & COLON-RAMOS, D. A. 2022. Presynaptic autophagy is coupled to the synaptic vesicle cycle via ATG-9. *Neuron,* 110**,** 824-840 e10.

YOUNG, A. R. J., CHAN, E. Y. W., HU, X. W., KOCH, R., CRAWSHAW, S. G., HIGH, S., HAILEY, D. W., LIPPINCOTT-SCHWARTZ, J. & TOOZE, S. A. 2006. Starvation and ULK1-dependent cycling of mammalian Atg9 between the TGN and endosomes. *Journal of Cell Science,* 119**,** 3888-3900.
