# Supplementary material for: The active zone protein Clarinet regulates synaptic sorting of ATG-9 and presynaptic autophagy
Source: PLoS Biol. 2023 Apr 13;21(4):e3002030. doi: 10.1371/journal.pbio.3002030 (PMC10101500; doi:10.1371/journal.pbio.3002030)
Supplement: Attachment — Submitted filename: Reponse to reviewers_final.docx [file pbio.3002030.s019.docx]

**REVIEWER COMMENTS**

**Reviewer #1:**

*The article "The active zone protein Clarinet regulates synaptic sorting of ATG-9 and presynaptic autophagy" by Xuan et al. shows that the active zone protein, Clarinet, regulates the activity-dependent sorting of ATG-9 at presynaptic terminals. This study provides new understanding to the field regarding how autophagosome proteins may be sorted at synapses, and suggests which proteins at the active zone and peri-active zone play key roles in this sorting. The authors addressed the major concerns raised by reviewers in the first version of the manuscript submitted to Dev Cell. I think the paper is suitable for publication in PLoS Biology after addressing the following minor comments:*

**1.      Supplemental figure 1C, D: Note that there was a problem with the images in this figure in one of our pdf viewers, such that only half of the image was visible. We were able to see the image in another viewer, but it may be worth a double check before publication.**

We have tried both illustrator and pdf viewers and the images open well in both viewers. We will work with the copy editors if additional changes to format are necessary for correct display of figures.

**2.      Figure 2I and 2J: The blue line is too thick and obscures structures near the plasma membrane, such as the active zone. The authors should make the line thinner.**

Now we make the blue line thinner and less opaque. We also added the original EM micrographs without annotations to Figure S3D-E.

**3.      Figure 2N and S3, which were added during revisions, are not adequately discussed in the text.

235 "Quantification of the presence of synaptic vesicles, dense core
236 vesicles and endosomal structures in the electron micrographs of cla-1(ola285)
237 and wild-type animals did not reveal major differences that could account for the
238 observed phenotypes of ATG-9 (Figure 2 I-N; Figure S3A-B).

The graph indicates that there are significant changes in synaptic vesicle and dense core vesicle number in the mutant, and it's unclear why the left and right neurons should have opposite phenotypes in the mutant. Does this discrepancy suggest that this experiment is not adequately powered to make quantitative conclusions? Are the cross-sections of similar area and if they are not, would it be better to normalize to area? The authors should discuss this result rather than glossing it over.**

The differences in AIYR (observed in Figure 2 for *cla-1(ola285)* mutants, also in S3 in endosomal area for wild type) are consistent with previous findings about asymmetry of AIY neurons (White et al., 1986), including gene expression (Hobert, 2014). Nonetheless, unlike for other genotypes we have previously examined by EM (like UNC-26/Synaptojanin) (Yang et al., 2022), the observed phenotypes do not reveal major differences that could account for the observed light-microscopy phenotypes of ATG-9. We now discuss this in the figure legend of Figure 2.

**4.      I do not agree with the author's interpretation of the immuno-EM data. They wrote:

255     Together,
256     our findings suggest that the ATG-9 phenotype in cla-1(ola285) results from
257     differences in the sorting of ATG-9-containing vesicular structures at the synapse.**

**I think the immuno-EM data confirms (and does not add much to) what they observe with the light microscopy which is that the ATG-9 positive vesicles are more clustered at presynaptic terminals in the cla-1 mutant compared to controls. I don't agree this data indicates that ATG-9 is sorted differently because they do not detect any morphological alterations that would suggest the nature of the ATG-9-associated compartment has changed in cla-1(ola285). These data only show that the distribution, not the sorting, of the ATG-9 vesicles is altered in cla-1(ola285) compared to controls. I think there are many explanations that could account for changes in distribution such as altered trafficking, mobility, recruitment to condensates etc. Though the experiments they mention later in the paper support the idea that the sorting of ATG-9 is disrupted, the altered distribution and disrupted sorting may be two separate phenomena.**

We agree with the reviewer that at this point in the manuscript using the word “distribution” is more accurate than “sorting”, and we edited the sentence accordingly. It now reads “Together, our findings indicate that the ATG-9 phenotype in *cla-1(ola285)* results from differences in the distribution of ATG-9-containing vesicular structures at the synapse " (Lines 242-244).

 **5.      Figure 8: I think this experiment really clearly shows that the C-terminus of CLA-1 localizes to the active zone while the N-terminus localizes to the peri-active zone. However, this experiment was not performed in the AIY interneurons. The authors should explain why they performed this single experiment in neurons of the posterior dorsal nerve cord instead of the AIY interneurons.**

Our studies in AIY were performed in the Zone 2 region which, based on our EM reconstructions, consists of three parallel active zone regions with overlapping periactive zone areas (Figure 2 and White et al, 1986). Due to the high density of presynaptic structures in the Zone 2 region, this makes nanoscale examination of CLA-1 localization challenging in that system. To be able to rigorously examine the relationship of CLA-1 to periactive regions, we chose a neuronal system in which active zone and periactive zone positions had been well characterized both by EM and light microscopy. The posterior dorsal nerve cord provided a good system for this characterization given previous studies that allowed us to cross reference our findings of CLA-1 position with known subsynaptic regions (Bai et al., 2010, White et al., 1986). We now clarify this choice in the methods (Lines 665-674).

**Reviewer #2:**

*This paper by Xuan et al follows up a recently published study from the same lab (Yang et al, 2022), which reports the biogenesis of ATG-9 containing vesicles from Golgi via AP-3 and how ATG-9 containing vesicles at AIY neuron synapses undergo activity-dependent exo-endocytosis.  Yang et al described a phenotype that in endocytosis mutants such as unc-26/synaptojanin, ATG-9 forms clathrin-rich subsynaptic foci.  Here, the authors identified, through genetic screening, a missense mutation in the active zone protein cla-1L that causes ATG-9 synaptic foci phenotype resembling unc-26 mutants.  They performed EM reconstruction of AIY synapses and found that overall presynaptic active zone and vesicles in the AIY neurons are largely unaltered in the cla-1L mutants. Immuno-EM of ATG-9::GFP show altered distribution in cla1L mutants.  They show CLA-1L localizes to a presynaptic subdomain periactive zone, different from other cla-1 protein isoforms. They carried out
extensive genetic interaction studies between cla-1l and AP1, AP2 and other endosomal sorting proteins.  These data led them to conclude that ATG-9 sorting in AIY synapse depends synaptic exo-endocytosis, as well as coordinated action of endosomal sorting machinery.  Finally, they show mis-sorting of ATG-9 in cla-1L mutants contributed to activity-induced autophagosome formation at synapses.

The manuscript presents a large body of work. The AIY synapse EM reconstruction is very impressive.  The analysis on CLA-1L and ola285 mutation is careful, and informative about synaptic protein subdomains.  While the message that CLA-1L mediated sorting of ATG-9 is dependent on synapse exo-endocytosis sounds similar to that in Yang et al, the differential effects of AP1, AP2, EPS15 and others in ATG-9 sorting make important contribution to understand synaptic endosomal network.  A missing puzzle is that the entire study did not touch on how exactly CLA-1-mediated ATG-9 sorting interface with unc-26 or endophilins, even though the similarity of cla-1L to unc-26 in ATG-9 localization phenotype was the starting point of the study and emphasized throughout the manuscript.  Some data and conclusions may be clarified and strengthened. 

Specific points:*

**1. Line 198-199, regarding the analysis on cla-1(wy1048): please clarify if the phenotype is only referring to ATG-9 synaptic foci.**

We have clarified that the phenotype refers to ATG-9 specifically (Line 185-186).

**2. Line 272-273 conclusion: 'These results demonstrate that the ATG-9 phenotype in cla-1(ola285) mutants results from defects in ATG-9 sorting upon ATG-9 exo-endocytosis.' It seems that this is based on double mutant analysis using synaptic vesicle exocytosis mutants.  How did author draw the link to ATG-9 exo-endocytosis?**

In (Yang et al., 2022) we performed several experiments in tissue culture cells and *in vivo* which were consistent with ATG-9-containing vesicles undergoing exo-endocytosis. Those findings in the Yang study were also consistent with published work demonstrating an accumulation of ATG-9 at the plasma membrane upon perturbation of dynamin-dependent endocytosis, and as detected by fluorescence microscopy (Puri et al., 2013, Popovic and Dikic, 2014, Puri et al., 2014). In this study we similarly demonstrated that mutants involved in synaptic vesicle exocytosis suppress the ATG-9 phenotype in *cla-1* mutants, and we interpret these findings to mean that the ATG-9 phenotype in *cla-1(ola285)* mutants, similar to the phenotype found in *unc-26/synaptojanin 1* mutants, results from defects in ATG-9 sorting upon ATG-9 exo-endocytosis. We have added our rationale in the text (Line 257-260).

**3. Line 297-298 and Figure 5M-N: the graphs need cla-1(ola285) for static comparison to support the enhanced expressivity of ATG-9 sorting defects.**

We have added *cla-1(ola285)* quantifications in Figure 5M-N for comparison.
 
**4. Figure S1D: the imaging seems to show GFP::CLA-1L(I5753N) protein. if so, please correct the panel label. This data would be better to be shown in the Figure 1 . The effect of the I5753N mutation on CLA-1L protein is striking.  The large size of CLA-1L protein makes biochemical studies difficult. Does protein modeling help shed some insights?**

We have changed the label of the images and moved them to Figure 1. CLA-1L consists mostly of an intrinsically disordered domain, so modeling did not result in additional insights. But based on the phenotype we hypothesize that the missense mutation results in degradation of CLA-1L isoform and a loss-of-function lesion. We modified the text to explain this (Lines 193-196).

**5. Pg 11-12, Figure 5E-H and Figure S2B: how does unc-26/synaptojanin and endophilin affect the co-segregation of ATG-9 and CHC-1 in cla-1(ola285)?  Similarly, please show data or comment on if AP3 has any role in cla-1L mediated ATG-9 sorting at AIY synapse.**

We did not specifically examine the ATG-9 or CHC-1 phenotypes in *cla-1;unc-26*  double mutants, as suggested. But given our analyses using other mutants involved in endocytosis (in this study), we hypothesize that the double mutant would similarly phenocopy the single *unc-26* and *cla-1* single mutants.

In (Yang et al, 2022) we found that putative null alleles for the three AP-3 complex subunits (apb-3/β3-adaptin, apm-3/μ3, apd-3/δ-adaptin) abnormally resulted in accumulation of ATG-9 at the Golgi, indicating that AP-3 acts upstream of ATG-9 transport and sorting at the synapse.

**6. Figure 6G-H: does sdpn-1; unc-11 show any difference from sdpn-1 alone?  does mutation in sdpn-1 suppress ATG-9 phenotypes in unc-11 and cla-1 to the same degree?**

The reviewer is correct in that there is a small, but statistically significant difference between the percentage of animals displaying ATG-9 puncta in *sdpn-1;unc-11* as compared to *sdpn-*1, consistent with *sdpn-*1 suppressing ATG-9 phenotypes in *unc-11* and *cla-1* to different degrees. We note however that quantification of the expressivity of the phenotype using the index (Fpeak-Ftrough)/Ftrough) for ATG-9::GFP (Figure 6H) did not uncover statistically significant differences between these genotypes. Because of the subtle nature of the phenotype and the focus of the paper, we did not elaborate on this difference.

**7. Figure 7I: does unc-101 suppresses cla-1 and unc-11 to same degree?**

The percentage of animals with ATG-9 puncta is slightly higher in *unc-101;unc-11* than in *unc-101; cla-1*, but that small difference is statistically significant (p<0.01 by two-tailed Fisher’s exact test). But similar to explained in point 6, we did not detect a difference in the expressivity of the phenotype.

**8. Figure 8: the data nicely show N- and C-terminal tagged CLA-1 protein subsynaptic difference in the nerve cord.  Since ATG-9 foci phenotype seems to be specific to AIY synapse zone 2, it would be appropriate to know how CLA-1 is localized in AIY, or revise the text to make it clear which synapses are and what the relevance is to ATG-9 sorting**

Our studies in AIY were performed in the Zone 2 region which, based on our EM reconstructions, consists of three parallel active zone regions with overlapping periactive zone areas (Figure 2 and White et al, 1986). This would make the relative distribution of CLA-1 domains hard to examine. To rigorously examine the relationship of CLA-1 to periactive zone regions, we chose a neuronal system in which active zone and periactive zone positions had been well characterized both by EM and light microscopy. The posterior dorsal nerve cord provided a good system or this characterization given previous studies that allowed us to cross reference our findings of CLA-1 position with subsynaptic regions (Bai et al., 2010) (White et al., 1986). We now clarify this choice in the methods (Lines 665-674).

Minor points:
 **9. Line 95: 'sort of', of is extra word?**

We have deleted " of ".

**10. Line 158:  consider to revise the sentence 'The phenotypes for synaptic vesicle proteins and ATG-9 differ in ola285 mutants' as it implies ola285 also affects synaptic vesicle proteins.**

We have deleted this sentence.

**11. Line 598, a typo: the cla-1L mutation is stated as nonsense mutation.**

We have changed "nonsense" to "missense".

**12. Figure 1M: it is unclear why there is no statistic analysis between cla-1(ola285)/cla-1(ok560) with respect to each homozygous mutation.**

“NS” (not significant) by two-tailed Fisher’s exact test has been marked in Figure 1M between cla-1(ola285)/cla-1(ok560) and both homozygous mutations.

**13. Consider to re-organize two supplemental figures to make contents cohesive.
Figure S1F-H is unrelated to A-E.
Figure S6 C-D is unrelated to A-B**

We have re-organized the two supplemental figures as below:

Original Figure S1F-H are now Figure S3A-C

Original Figure S6 C-I are now Figure S7A-G

**14. Figure S5: the cartoon diagrams are nice, but it is unclear what 'OR' is intended for.**

We simplified the diagrams by keeping only one shape to symbolize the endocytic intermediates containing ATG-9.

**15. line 1280: 'insets it' means 'insets at'**

Typo has been corrected.

**Reviewer #3:**

*In this manuscript, Xuan et al performed an unbiased genetic screen (by mutagenesis) in C. elegans to identify proteins involved in the autophagosome cycle, and specifically of vesicles incorporating ATG-9. Their main finding is that the active zone protein Clarinet is specifically involved in the recycling of ATG-9 containing vesicles, but does not appear to participate in the recycling of synaptic vesicles. They use mutants of proteins involved in sequential steps of vesicle sorting to illustrate the dependence of cycling and sorting of ATG-9 on clarinet. Specifically, they show that disrupting bulk endocytosis antagonizes the clarinet-dependent deficit in ATG-9 localization, while disruption of sorting of endocytosis intermediates enhances it. Likewise, inhibiting exocytosis also antagonizes the clarinet dependent effect on ATG-9 cycling. Also, ATG-9 colocalizes with clathrin when sorting of endocytosis products is deficient.  The authors fuse GFP to both
ends of clarinet to show that it extends from the active zone to the endocytosis-active peri-active zone. In this context, they illustrate a cooperative relationship between clarinet and the EPS15/Intersectin1 endocytic scaffolding that couples between active zone exocytosis and periactive zone endocytosis. Finally, they show that the mutation in clarinet reduces activity-dependent autophagosome formation, based on imaging of LGG-1.
This manuscript is a revised version, after it was referred to PLOS biology from Dev Cell. The authors addressed much of the concerns raised by the previous reviewers. Furthermore, they clearly differentiated between the novel findings of this study (the involvement of clarinet in the sorting of ATG-9 containing vesicles) and the findings of a previous study already published by the same group in Neuron concerning the involvement of ATG-9 in SV autophagy.
I don't have major concerns about the manuscript, and I find that it contributes significantly to the advancement of our knowledge on this important topic. Furthermore, the manuscript is written clearly, and the logic behind the experiments is explained amply.
I do have a few questions for the authors:*

**1.**      **The authors indicate that clarinet is involved in activity-dependent autophagy and sorting of ATG-9, but that baseline autophagy is not affected. On this basis, wouldn't they expect that the ATG-9 phenotype in clarinet mutants should resolve over extended periods of time? I am puzzled by the fact that the authors observe steady-state ATG-9 puncta, but they indicate that only activity-dependent autophagy is affected by the clarinet mutation. In this context, is there a difference between the number of LGG1 puncta at 20C between WT and clarinet mutants? The authors provided a statistical analysis only for the effect of the shift in temperature but not the effect of the ola285 mutation itself (as far as I could tell).**

The ATG-9 phenotype in *cla-1* mutants was observed at 20C, but the LGG-1 phenotype was only observed at 25C, when the activity state of the neurons is elevated. Our interpretation of this data is that we are assaying flux states, both of ATG-9 and for autophagosome formation. In other words, we believe that the *cla-1* mutation results in defects in ATG-9 sorting that slow down its normal deployment to facilitate synaptic autophagy, and that by “stress-testing” the system via the increase in activity-dependent autophagy we are revealing the requirement of the normal states of flux in ATG-9 sorting. We now better explain this in the text (Line 426-431). We will also like to clarify that the difference in lgg-1 number between WT and *cla-1* at 25C in Fig 9D was statistically significant (p<0.01).

**2.      In this respect, do the authors have any EM data relating directly to the autophagosomes themselves? I.e., in their diagrams the authors allude to recycling endosomes or other intermediates and to autophagosomes that should include ATG-9, but do not visualize those directly, at least in the EM data. According to the diagrams (Figure 10 and supplementary figure 5), it could be expected to observe such ATG-9 labeled intermediates, especially in clarinet mutants.**

Autophagosomes are short-lived structures in *C. elegans* neurons, and in wild type animals, or in mutants that affect autophagosome formation, we have not been able to detect them in EM micrographs. We have only detected them in mutants that affect flux by preventing autophagosome breakdown (Hill et al., 2019). The phenotype we observe by EM in *cla-1* mutants regards the distribution of ATG-9 containing vesicles. We note that clusters of ATG-9-containing vesicular structures have also been observed in vertebrate cells defective for autophagy (Olivas et al., 2022), suggesting that clustering of ATG-9 vesicles, such as those seen for *cla-1* mutants, might be a hallmark of defective autophagy (we elaborate on this in the Discussion).

In response to the reviewer’s comment and to better represent this in the model of Fig 10, we modified our original model and replaced the big endosome-like structure with a cluster of ATG-9-containing vesicles.

**3.      The authors claim that clarinet does not affect synaptic vesicle cycling, based on their examination of Rab3 distributions in WT and ola285 mutants. Do they have functional readouts to substantiate this very important claim (besides immunofluorescence analyses)?**

We want to first clarify that the active zone protein CLA-1L is necessary for synaptic vesicle clustering in various neurons, including AIY ((Xuan et al., 2017); in that manuscript we also examined functional requirements of *cla-1L* at the synapse by electrophysiology and aldicarb assays) and concluded that CLA-1L is specifically required for proper synaptic function during repeated stimulations. Our claim is not that CLA-1L does not affect synaptic vesicles, but that the requirement of CLA-1L on synaptic vesicle recycling is different from its requirement in ATG-9 sorting, because it results in different phenotypes for ATG-9 and synaptic vesicle markers distributions. Thus, *cla-1(ola285)* allowed us to separate the pathways sorting synaptic vesicles and ATG-9, and further probe the underlying mechanisms for ATG-9 sorting (we elaborate on this in the Discussion).

**4.      I could not understand why the authors observed a difference between ola285-AIYL and ola285-AIYR in figure 2. Is the mutation targeted to one but not the other?**

The mutations affect the whole organism. The differences in AIYR (observed in Figure 2 for *cla-1(ola285)* mutants, also in S3 in endosomal area for wild type) are consistent with previous findings about asymmetry of AIY neurons (White et al., 1986), including gene expression (Hobert, 2014). Nonetheless, unlike for other genotypes we have previously examined by EM (like UNC-26/Synaptojanin) (Yang et al., 2022), the observed phenotypes do not reveal major differences that could account for the observed light-microscopy phenotypes of ATG-9. We now discuss this in the figure legend of Figure 2.

**Reviewer #4:**

*The manuscript by Xuan et al. examines the novel role of active zone scaffold protein Clarinet/CLA-1 in mediating the sorting of autophagic lipid scramblase protein ATG-9 at presynapses. Using unbiased forward genetic screens of presynaptic ATG-9 localization in the AIY neuron of C. elegans, the authors identify the long isoform of CLA-1 as a regulator of ATG-9 presynaptic distribution/sorting. Through a series of genetic experiments combined with confocal and electron microscopy, the authors suggest a model in which ATG-9 is endocytosed and sorted into endocytic intermediates via proteins including Syndapin-1 and the AP-1 complex, while CLA-1L and the AP-2 and AP180 complexes sort ATG-9 out of these endocytic intermediates and into exocytosis-ready vesicles. Additionally, the authors show that activity-induced autophagosome formation at presynapses in AIY are impaired in CLA-1L mutants. 

While not providing a complete mechanism, this manuscript does offer strong evidence for a role for CLA-1L as a mediator of ATG-9 sorting at synapses, and requires only minor clarifications/revisions:*

**1.      Confocal imaging parameters: The authors explain in the methods section that ATG-9::GFP was imaged using different exposure settings between WT and cla-1(ola285) animals. While the decision to use these different parameters is explained in the author response to a previous reviewer, all validation should be clearly communicated in a supplemental figure that includes representative images of both genotypes at both exposures, and with comparisons of the index of ATG-9 puncta between all four conditions. Providing this information will help more convincingly make the argument that the relationship between the indices of ATG-9 distribution remains consistent, even when imaging at different exposures between genotypes.**

We have added the images in Figure S1 (E-I) as suggested by the reviewer. We added the quantification for *cla-1(ola285)* at lower exposure setting to show that the difference in the index between WT and *cla-1(ola285)* does not depend on exposure settings (Figure S1I). We did not include *cla-1(ola285)* regular setting for quantification because the signal was saturated and does not capture the dynamic range necessary for the correct quantification of the phenotype (which is also part of the justification for using the lower exposure with this genotype). This is now also explained in the figure legend for the new supplementary figure.

**2.      In Figure 2N, each point is reported to represent a single section. Further information about the number of neurons (n) represented in each condition should be reported. Additionally, the significant difference between AIY-right in cla-1(ola285) mutants and the other genotypes is reported but not commented on in the text. Is this finding relevant to the fluorescence images presented, which are not labeled as AIY-left or -right in any figures?**

We now include the information about the number of worms in the figure legends. We also comment on the difference of synaptic vesicles and dense core vesicles in AIYR in *cla-1(ola285)* mutants in Figure 2 legend. Briefly, the differences in AIYR (observed in Figure 2 for *cla-1(ola285)* mutants, also in S3 in endosomal area for wild type) are consistent with previous findings about asymmetry of AIY neurons (White et al., 1986), including gene expression (Hobert, 2014). Nonetheless, unlike for other genotypes we have previously examined by EM (like UNC-26/Synaptojanin) (Yang et al., 2022), the observed phenotypes do not reveal major differences that could account for the observed light-microscopy phenotypes of ATG-9.

**3.      In the quantification of Supplemental Figure 3, the average endosome area is displayed for each cross section, but further details about the total number and volume of endosomes present in each genotype would be helpful to understand whether there is any effect on the endosomal pathway. Indeed, in the model provided in Figure 10, the intracellular endocytic intermediates are displayed in two forms (endosomes or vesicles) with "or" written between them. This makes the model more difficult to follow, and the information gleaned from the multiple EM reconstructions in this work should offer some insight into the morphology of these intermediates.**

We now include the total endosome count in AIY of wild type and *cla-1* mutants (Figure S3H). The relationship between the area measurements and the volume is the thickness of the section, which is 40nm. Because this number is constant for all sections, the volume is directly proportional to all our area measurements. We now explain this in the methods (lines 696-699). We modified our model in Figure 10 and replaced the big endosome-like structure with a cluster of vesicles containing ATG-9, which better aligns with our observations by EM.

**4.      In Figure 8, it is confusing and unclear why the overall colocalization between CLA-1 and APT-4 is so low, and only being a C. elegans researcher and digging deep into the strain information was it possible to understand that APT-4 is only being expressed in a subset of neurons in the dorsal nerve cord (under the Punc-129 promoter), while CLA-1::GFP-FLP-on is expressed pan-neuronally (through Prab-3 > Flpase) and GFP::CLA-1 is constitutively expressed pan-neuronally. A better experimental design would be to drive APT-4 from a pan-neuronal promoter when comparing with GFP::CLA-1L strains, in order to compare colocalization of CLA-1L with APT-4 in all neurons along the dorsal nerve cord. Additionally, the C-terminally tagged CLA-1::GFP-FLP-on could be expressed in the same subset of neurons as APT-4 by expressing flippase driven by Punc-129. At the minimum, further clarification of the fact that APT-4 is expressed in only a subset of neurons in the dorsal nerve cord, and
what this means for the colocalization analysis, should be provided in the text. However, the localization of CLA-L is a central point in the paper, and the proposed experiments are quite simple, and would more convincingly demonstrate the point being made, especially to a non-worm reader.**

We appreciate the comment and the thoughtfulness behind the experimental suggestion. The two main authors are no longer in the lab, complicating completion of suggested experiments. Nonetheless, we have now clarified in the text as suggested (Lines 665-674).

**5.      At first glance in Figure 9A-C, the inset boxes appear to be additional regions within the image, rather than magnifications of the boxed regions below. Perhaps boxing the magnified insets in a solid line would be clearer.**

We have modified Figure 9A-C as suggested.

BAI, J. H., HU, Z. T., DITTMAN, J. S., PYM, E. C. G. & KAPLAN, J. M. 2010. Endophilin Functions as a Membrane-Bending Molecule and Is Delivered to Endocytic Zones by Exocytosis. *Cell,* 143**,** 430-441.

HILL, S. E., KAUFFMAN, K. J., KROUT, M., RICHMOND, J. E., MELIA, T. J. & COLON-RAMOS, D. A. 2019. Maturation and Clearance of Autophagosomes in Neurons Depends on a Specific Cysteine Protease Isoform, ATG-4.2. *Developmental Cell,* 49**,** 251-+.

HOBERT, O. 2014. Development of left/right asymmetry in the Caenorhabditis elegans nervous system: from zygote to postmitotic neuron. *Genesis,* 52**,** 528-43.

OLIVAS, T. J., WU, Y., YU, S., LUAN, L., CHOI, P., NAG, S., DE CAMILLI, P., GUPTA, K. & MELIA, T. J. 2022. ATG9 vesicles comprise the seed membrane of mammalian autophagosomes. *bioRxiv***,** 2022.08.16.504143.

POPOVIC, D. & DIKIC, I. 2014. TBC1D5 and the AP2 complex regulate ATG9 trafficking and initiation of autophagy. *EMBO Rep,* 15**,** 392-401.

PURI, C., RENNA, M., BENTO, C. F., MOREAU, K. & RUBINSZTEIN, D. C. 2013. Diverse autophagosome membrane sources coalesce in recycling endosomes. *Cell,* 154**,** 1285-99.

PURI, C., RENNA, M., BENTO, C. F., MOREAU, K. & RUBINSZTEIN, D. C. 2014. ATG16L1 meets ATG9 in recycling endosomes: additional roles for the plasma membrane and endocytosis in autophagosome biogenesis. *Autophagy,* 10**,** 182-4.

WHITE, J. G., SOUTHGATE, E., THOMSON, J. N. & BRENNER, S. 1986. The structure of the nervous system of the nematode Caenorhabditis elegans. *Philos Trans R Soc Lond B Biol Sci,* 314**,** 1-340.

XUAN, Z., MANNING, L., NELSON, J., RICHMOND, J. E., COLON-RAMOS, D. A., SHEN, K. & KURSHAN, P. T. 2017. Clarinet (CLA-1), a novel active zone protein required for synaptic vesicle clustering and release. *Elife,* 6.

YANG, S., PARK, D., MANNING, L., HILL, S. E., CAO, M., XUAN, Z., GONZALEZ, I., DONG, Y., CLARK, B., SHAO, L., OKEKE, I., ALMORIL-PORRAS, A., BAI, J., DE CAMILLI, P. & COLON-RAMOS, D. A. 2022. Presynaptic autophagy is coupled to the synaptic vesicle cycle via ATG-9. *Neuron,* 110**,** 824-840 e10.
